# Biomimetic rehabilitation of a structurally compromised endodontically treated tooth using deep margin elevation and polyethylene fiber–reinforced post and core: a case report

**DOI:** 10.3389/fdmed.2026.1873841

**Published:** 2026-07-02

**Authors:** Derek Shaji Pious, Chitharanjan M. Shetty, Maria Anna Geevarghis, Sunheri Bajpe, Rashi Shroff

**Affiliations:** Department of Conservative Dentistry and Endodontics, AB Shetty Memorial Institute of Dental Sciences, NITTE Deemed to be University, Mangalore, India

**Keywords:** biomimetic rehabilitation, case report, deep margin elevation, doughnut technique, endodontically treated tooth, immediate dentin sealing, polyethylene fiber post, ribbond

## Abstract

**Background:**

Restoration of structurally compromised endodontically treated teeth with extensive coronal destruction and subgingival margins remains a significant restorative challenge. Contemporary biomimetic restorative approaches emphasize preservation of the remaining tooth structure through adhesive reinforcement strategies that improve biomechanical performance while minimizing additional radicular dentin removal.

**Case presentation:**

This case report describes the biomimetic rehabilitation of a previously root canal–treated maxillary canine with severe coronal destruction and subgingival palatal and proximal margins. Following laser gingivectomy and margin exposure, complete caries excavation was performed using caries detector dye guidance, followed by immediate dentin sealing using Prime & Bond NT (Dentsply DeTrey GmbH, Konstanz, Germany). Deep margin elevation was carried out using the doughnut technique with SDR Flow + Flowable Composite (Dentsply Sirona, Charlotte, NC, USA). Conservative post-space preparation was subsequently performed, and a polyethylene fiber–reinforced post and composite core buildup were completed using dual-cure resin cement under adhesive protocol. Definitive rehabilitation was achieved using a monolithic zirconia crown fabricated from Cercon XT zirconia (Dentsply Sirona, Bensheim, Germany), conditioned according to the APC (air-particle abrasion, primer application, and composite resin cementation) protocol prior to adhesive cementation.

**Outcome:**

Clinical and radiographic follow-up at one month demonstrated satisfactory esthetic and functional rehabilitation with healthy periodontal response, satisfactory marginal adaptation, and absence of clinical symptoms.

**Conclusion:**

Within the limitations of a single case report and short-term follow-up, polyethylene fiber–reinforced post and core systems combined with adhesive biomimetic restorative protocols such as immediate dentin sealing and deep margin elevation may represent a conservative treatment option for rehabilitation of structurally compromised endodontically treated teeth with compromised ferrule and subgingival defects.

## Introduction

Restoration of structurally compromised endodontically treated teeth (ETT) remains a significant clinical challenge, particularly in cases with extensive loss of coronal tooth structure and subgingival extension of cavity margins. Although successful endodontic therapy eliminates pulpal pathology, the long-term prognosis of ETT largely depends on the quality of the post-endodontic restoration and preservation of the remaining tooth structure. Excessive loss of dentin, reduced ferrule height, and compromised marginal integrity increase the susceptibility of such teeth to biomechanical failure and catastrophic fracture (1–3). The absence or inadequacy of ferrule in structurally compromised teeth significantly reduces fracture resistance and complicates restorative rehabilitation (3).

Conventional post systems, especially metallic and rigid prefabricated posts, have been associated with unfavorable stress distribution and increased risk of irreparable root fractures because of the mismatch in elastic modulus between the post material and dentin (2). Recent biomimetic restorative concepts therefore advocate minimally invasive adhesive strategies that reinforce the remaining tooth structure while preserving radicular dentin (4, 5). Polyethylene fiber–reinforced post and core systems have gained considerable attention owing to their dentin-like biomechanical behavior, multidirectional reinforcement, and ability to distribute functional stresses more evenly along the root structure (6–8). These fibers exhibit a crack-stopping mechanism and are associated with more favorable and repairable fracture patterns when compared with conventional post systems (1, 9).

Management of subgingival defects in structurally compromised teeth presents an additional restorative challenge. Traditionally, surgical crown lengthening was considered the preferred approach for gaining access to deep cervical margins; however, it may compromise periodontal support and esthetics (10). Contemporary adhesive restorative protocols such as deep margin elevation (DME) have emerged as conservative alternatives that relocate deep cervical margins coronally, thereby improving isolation, bonding procedures, and restorative adaptation while preserving periodontal health (11–14, 30). Furthermore, the doughnut technique allows pre-endodontic buildup of severely damaged teeth while maintaining access to the root canal system, thereby improving structural integrity and rubber dam isolation during treatment (15–18).

Immediate dentin sealing (IDS) has also been advocated as an important step in adhesive rehabilitation because it improves bond strength, preserves dentin substrate integrity, and reduces microleakage and postoperative sensitivity (19). Adhesive cementation protocols combined with fiber-reinforced post systems contribute to the formation of a biomechanically integrated restorative complex capable of enhancing fracture resistance and long-term clinical performance (20, 21).

Recently, zirconia crowns have become increasingly popular for definitive restoration of endodontically treated teeth because of their favorable mechanical properties, esthetics, and durability (22–24). Surface conditioning protocols such as the APC concept, involving air-particle abrasion and primer application before adhesive cementation, have further improved bonding reliability and clinical longevity of zirconia restorations (23).

The present case report describes the biomimetic rehabilitation of a structurally compromised maxillary canine with subgingival margins using laser-assisted crown exposure, deep margin elevation with the doughnut technique, immediate dentin sealing, and a polyethylene fiber–reinforced post and core followed by definitive zirconia crown restoration.

## Case report

### Patient information

A 45-year-old female patient reported to the Department of Conservative Dentistry and Endodontics with the chief complaint of a fractured upper left anterior tooth. The patient had undergone root canal treatment with respect to tooth 23 one year previously. The patient did not report any relevant medical history, systemic illness, allergies, or history of parafunctional habits.

### Clinical findings

**Figure 1:**
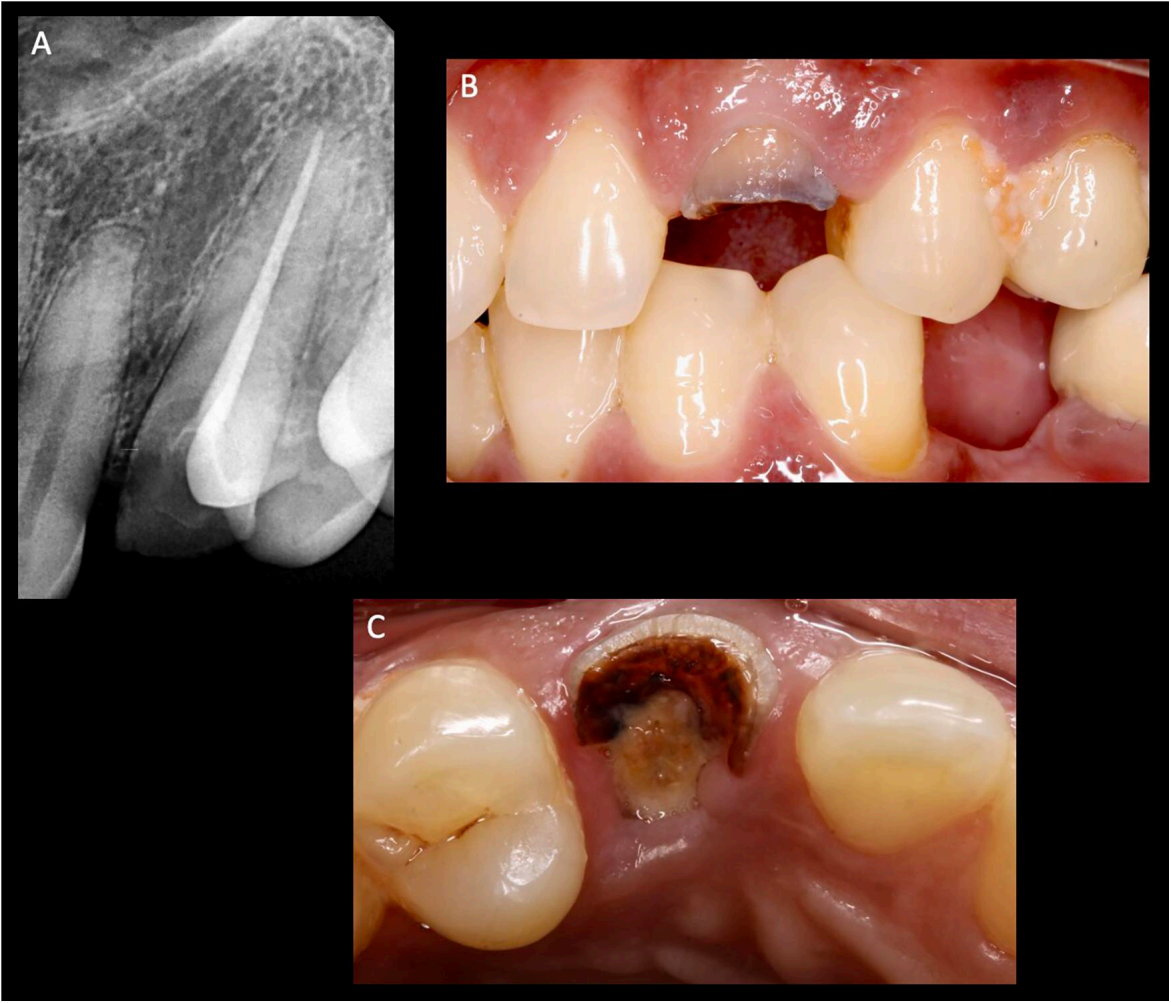
Preoperative assessment of tooth 23. **(A)** Preoperative intraoral periapical radiograph showing a previously root canal–treated tooth with satisfactory obturation and absence of periapical pathology. **(B)** Preoperative clinical view demonstrating extensive coronal destruction of tooth 23. **(C)** Occlusal view showing the structurally compromised tooth with palatal involvement.

Clinical examination revealed approximately 75%–80% loss of coronal tooth structure involving tooth 23, with an estimated ferrule height of 1–1.5 mm on the buccal aspect, less than 1 mm on the proximal surfaces, and virtually absent ferrule on the palatal aspect. The palatal and proximal margins extended approximately 1–2 mm subgingivally (Figures 1B,C). The tooth was asymptomatic, with no tenderness on percussion or palpation. Periodontal examination revealed no mobility or periodontal pocketing associated with the involved tooth.

Radiographic examination demonstrated a satisfactorily obturated root canal with no evidence of periapical pathology or root resorption (Figure 1A).

### Diagnostic assessment

Based on the clinical and radiographic findings, the tooth was diagnosed as previously treated with normal apical tissues according to the American Association of Endodontists (AAE) 2009 diagnostic criteria. Considering the satisfactory endodontic status and absence of periapical pathology, preservation of the tooth through adhesive biomimetic rehabilitation was planned. Since the existing root canal treatment was radiographically satisfactory and the tooth was asymptomatic, nonsurgical retreatment was not considered necessary.

### Therapeutic intervention

**Figure 2:**
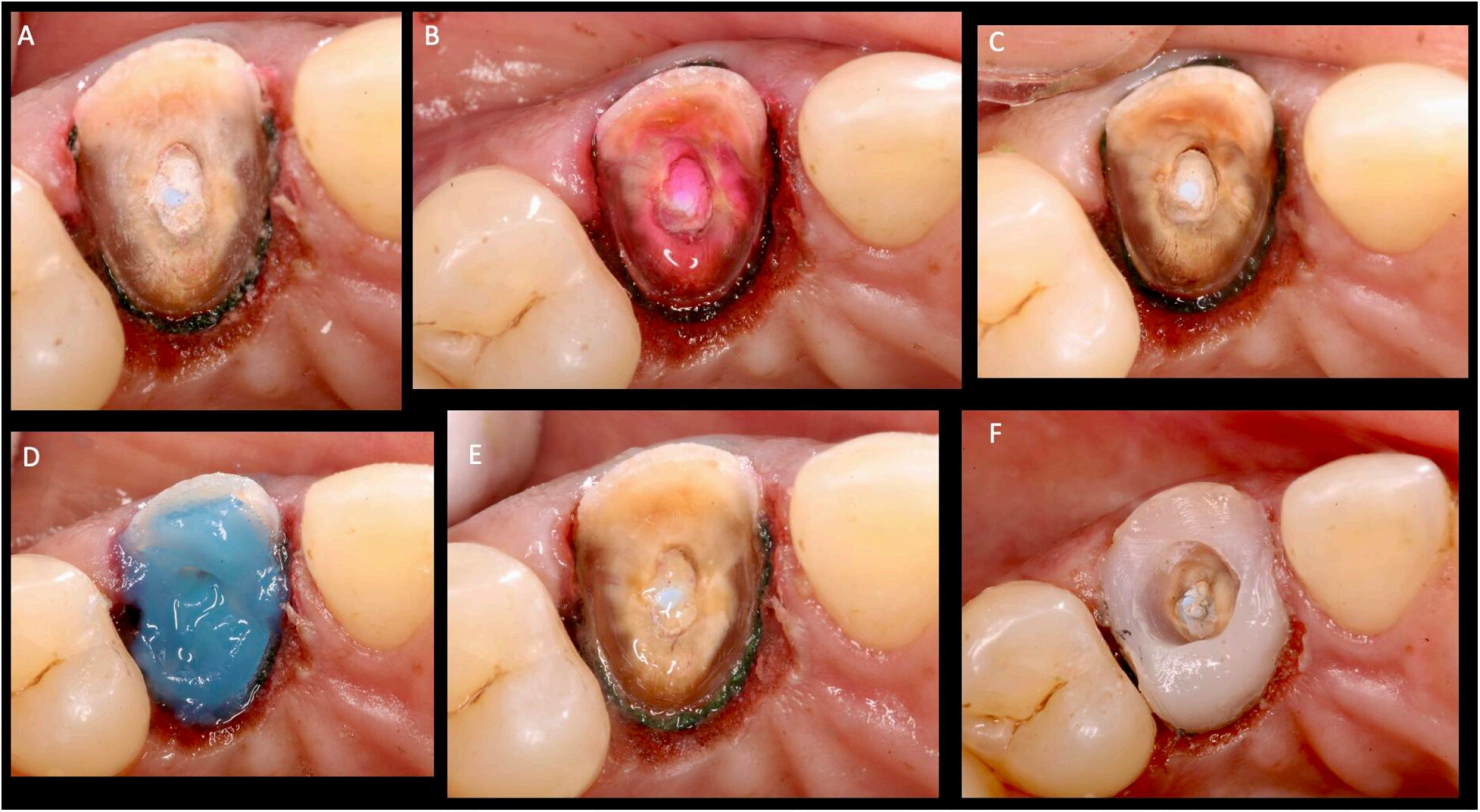
Management of subgingival margins and adhesive foundation buildup. **(A)** Initial caries excavation followed by laser gingivectomy and placement of retraction cord for margin exposure. **(B)** Application of caries detector dye for assessment of infected dentin. **(C)** Complete caries excavation followed by immediate dentin sealing (IDS). **(D)** Acid etching procedure. **(E)** Application of bonding agent. **(F)** Deep margin elevation using the doughnut technique with SDR Flow + Flowable Composite (Dentsply Sirona, Charlotte, NC, USA).

Initial caries excavation was performed to remove superficial infected dentin. Owing to the subgingival extension of the proximal and palatal margins, laser gingivectomy was performed using a 940 nm diode laser operating at 1.0 W in continuous mode to expose the cervical margins and facilitate restorative isolation, followed by placement of a retraction cord (Figure 2A).

Caries detector dye was subsequently applied to identify residual infected dentin (Figure 2B), followed by complete caries excavation. Immediate dentin sealing (IDS) was then performed using Prime & Bond NT (Dentsply DeTrey GmbH, Konstanz, Germany) after complete removal of carious dentin (Figure 2C).

Deep margin elevation (DME) was planned to relocate the deep cervical margins coronally and improve accessibility for adhesive restorative procedures. Selective enamel etching was performed using 37% phosphoric acid (Figure 2D), followed by application of Prime & Bond NT (Dentsply DeTrey GmbH, Konstanz, Germany) (Figure 2E). A pre-endodontic buildup using the doughnut technique was carried out with SDR Flow + Flowable Composite (Dentsply Sirona, Charlotte, NC, USA) while maintaining access to the root canal system (Figure 2F).

**Figure 3:**
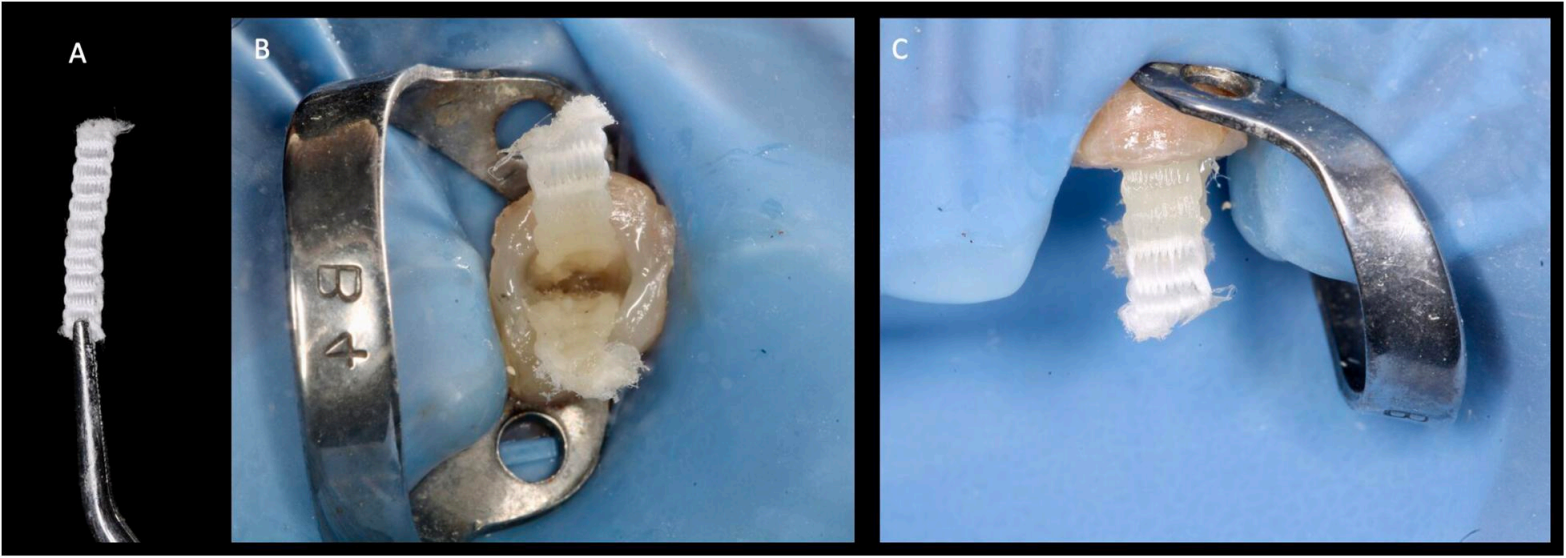
Post-space preparation and adhesive protocol for polyethylene fiber–reinforced post placement. **(A)** Rubber dam isolation of tooth 23. **(B)** Intraoral periapical radiograph showing post-space preparation for polyethylene fiber post placement. **(C)** Etching of the post space. **(D)** Application of bonding agent within the post space.

Following completion of the adhesive foundation buildup, rubber dam isolation was achieved (Figure 3A). Post-space preparation was performed using a size #1 post drill to an estimated depth of approximately 5–6 mm within the root canal while preserving approximately 10–11 mm of apical gutta-percha to maintain an adequate apical seal (Figure 3B). The prepared post space represented approximately one-third of the total root length. The prepared post space was etched with phosphoric acid (Figure 3C), followed by application of Prime & Bond NT (Dentsply DeTrey GmbH, Konstanz, Germany) within the canal space (Figure 3D).

**Figure 4:**
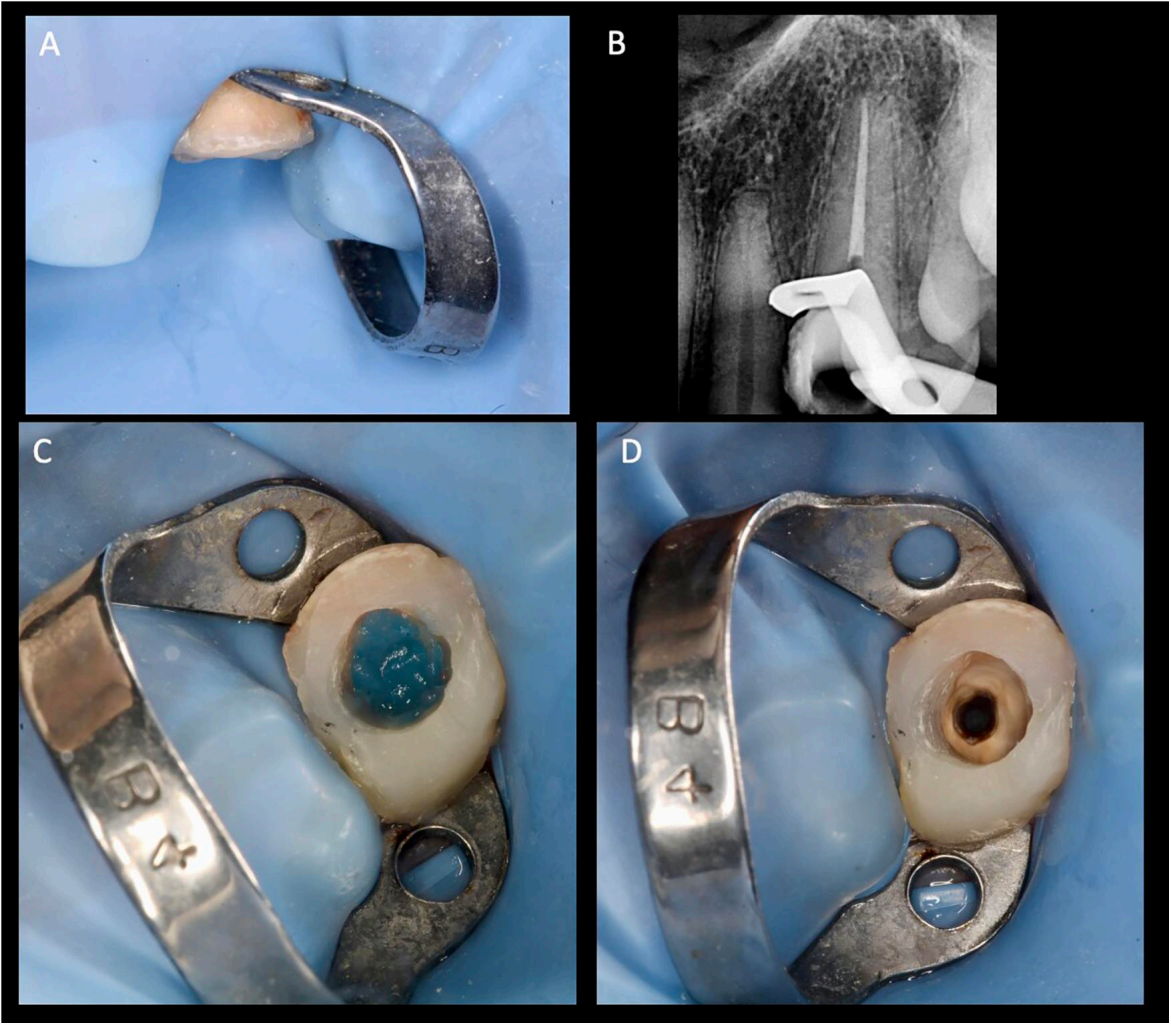
Placement of polyethylene fiber–reinforced post system. **(A)** Ribbond THM polyethylene fiber ribbon (Ribbond Inc., Seattle, WA, USA) used for reinforcement. **(B)** Adaptation and compaction of the polyethylene fiber ribbon into the prepared post space using Calibra Dual Cure Resin Cement (Dentsply Sirona, Charlotte, NC, USA). **(C)** Buccal view following placement of the polyethylene fiber post system.

**Figure 5:**
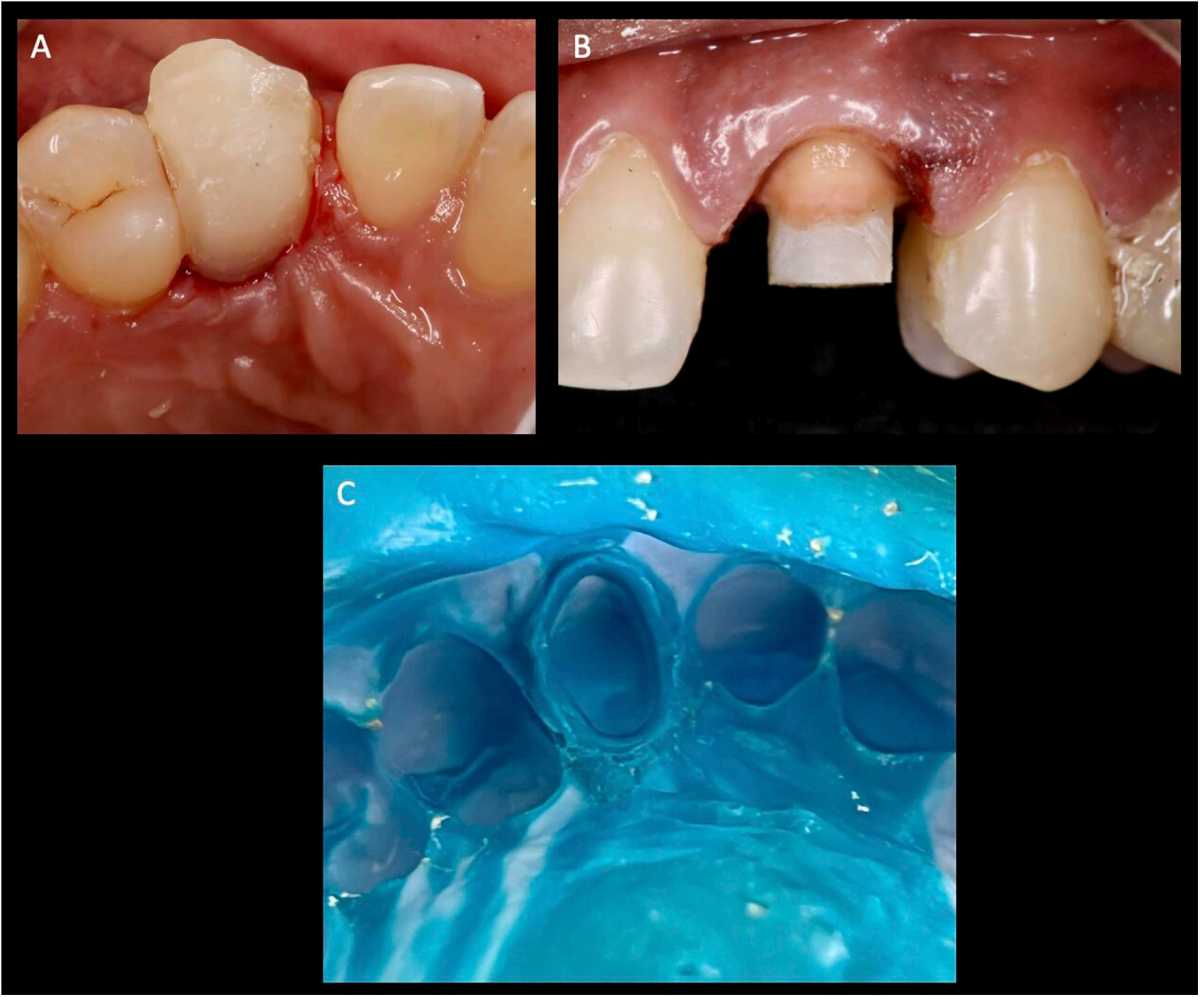
Core buildup and tooth preparation procedures. **(A)** Core buildup using Neo Spectra ST composite resin (Dentsply Sirona, Charlotte, NC, USA) following placement of the polyethylene fiber post. **(B)** Tooth preparation for zirconia crown restoration. **(C)** Putty-wash impression procedure for fabrication of the definitive restoration.

Ribbond THM polyethylene fiber ribbon (Ribbond Inc., Seattle, WA, USA) was measured according to the dimensions of the prepared post space and cut accordingly. The fiber was wetted with adhesive resin prior to insertion. Calibra Dual Cure Resin Cement (Dentsply Sirona, Charlotte, NC, USA) was injected into the canal space, and the polyethylene fiber ribbon was then carefully adapted and compacted into the prepared post space using a condenser to ensure intimate adaptation along the canal walls (Figures 4A–C). Composite resin core buildup was subsequently completed using Neo Spectra ST composite resin (Dentsply Sirona, Charlotte, NC, USA) to restore the coronal structure and provide support for definitive prosthetic rehabilitation (Figure 5A).

Tooth preparation for a full-coverage monolithic zirconia crown fabricated from Cercon XT zirconia (Dentsply Sirona, Bensheim, Germany) was then performed following biomechanical and esthetic principles. (Figure 5B). A putty-wash impression was made and sent for laboratory fabrication of the definitive zirconia crown (Figure 5C).

**Figure 6:**
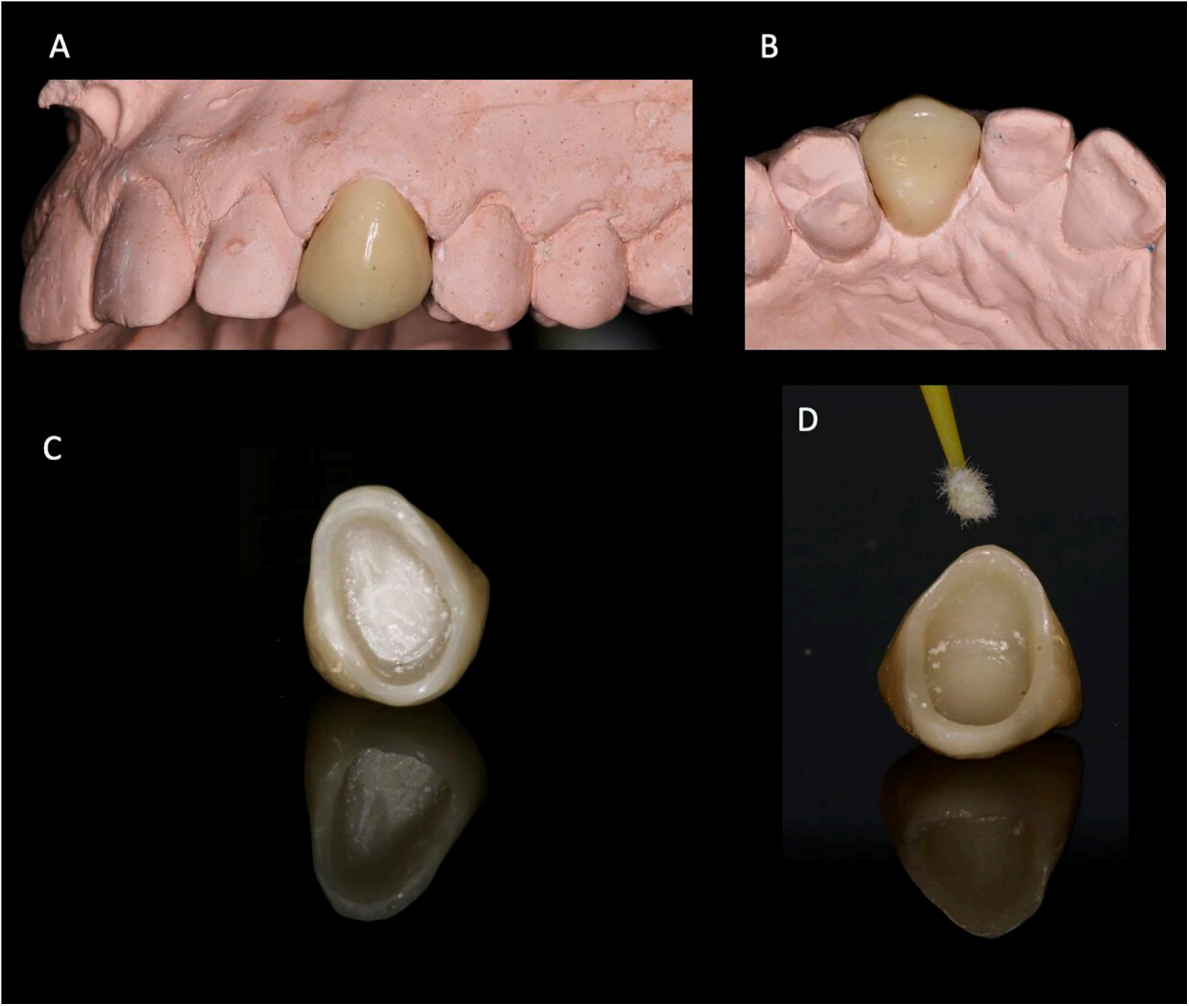
Zirconia crown fabrication and surface treatment procedures. **(A)** Buccal view of the fabricated zirconia crown. **(B)** Intaglio/occlusal view of the zirconia crown. **(C)** Air-particle abrasion of the zirconia surface. **(D)** Application of Monobond Plus (Ivoclar Vivadent, Schaan, Liechtenstein) according to the APC (air-particle abrasion, primer application, and composite resin cementation) protocol prior to cementation.

The fabricated zirconia crown was evaluated clinically for marginal adaptation and esthetics (Figures 6A,B). Prior to cementation, the intaglio surface of the zirconia crown was treated according to the APC (air-particle abrasion, primer application, and composite resin cementation) involving air-particle abrasion (Figure 6C) followed by application of Monobond Plus (Ivoclar Vivadent, Schaan, Liechtenstein) (Figure 6D). The crown was then adhesively cemented using Calibra Dual Cure Resin Cement (Dentsply Sirona, Charlotte, NC, USA).

### Follow-up and outcomes

**Figure 7:**
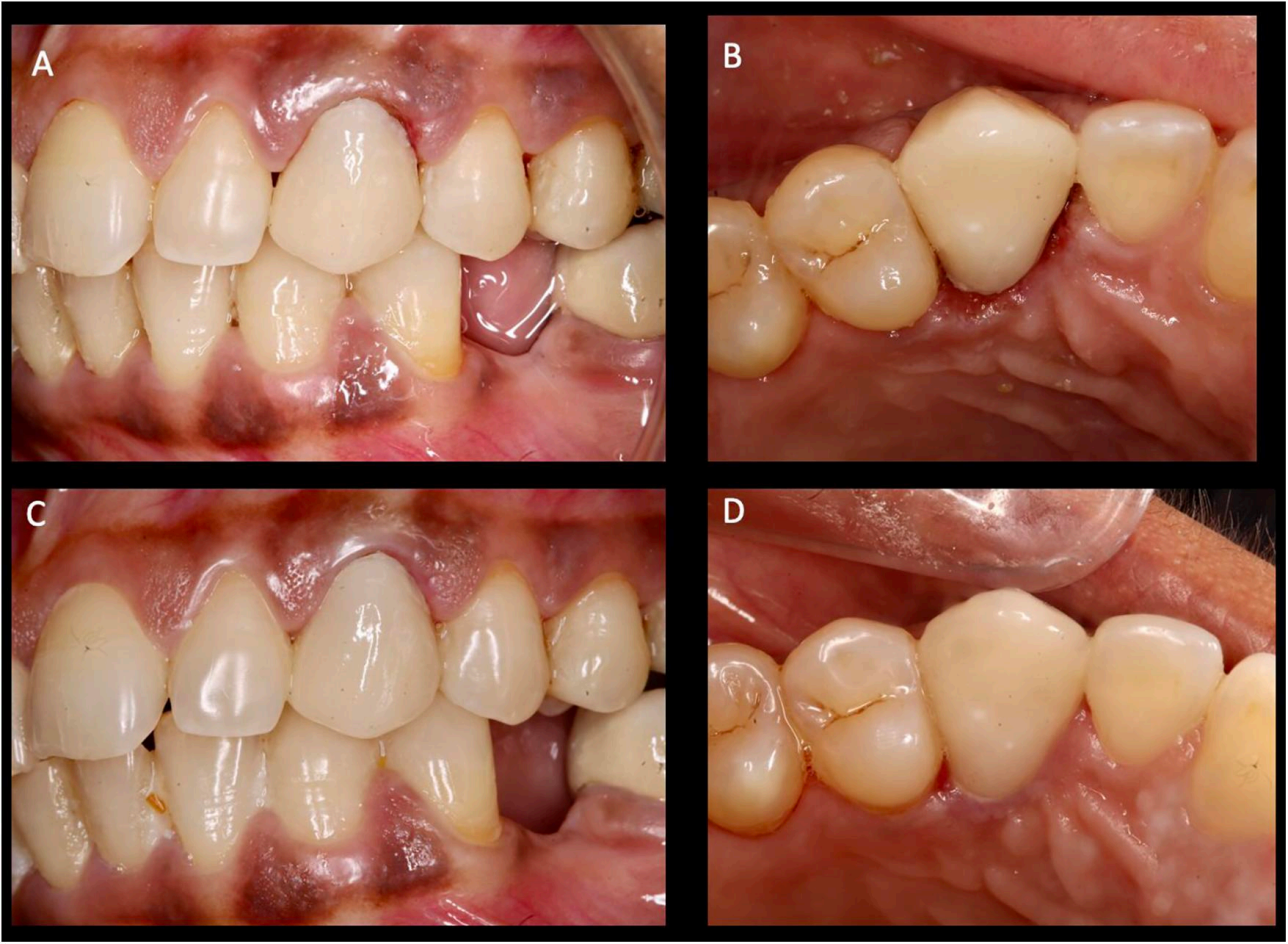
Cementation and follow-up evaluation. **(A)** Immediate postoperative buccal view after zirconia crown cementation. **(B)** Immediate postoperative occlusal view. **(C)** One-month follow-up buccal view demonstrating satisfactory gingival healing and restoration adaptation. **(D)** One-month follow-up occlusal view.

Immediate postoperative evaluation demonstrated satisfactory marginal adaptation, esthetics, and occlusal harmony (Figures 7A,B). At the one-month follow-up, the patient was asymptomatic, and clinical examination revealed satisfactory gingival healing and healthy periodontal response around the definitive restoration (Figures 7C,D). Clinical follow-up at one month demonstrated satisfactory periodontal healing, marginal adaptation, and functional rehabilitation of the restored tooth.

**Figure 8:**
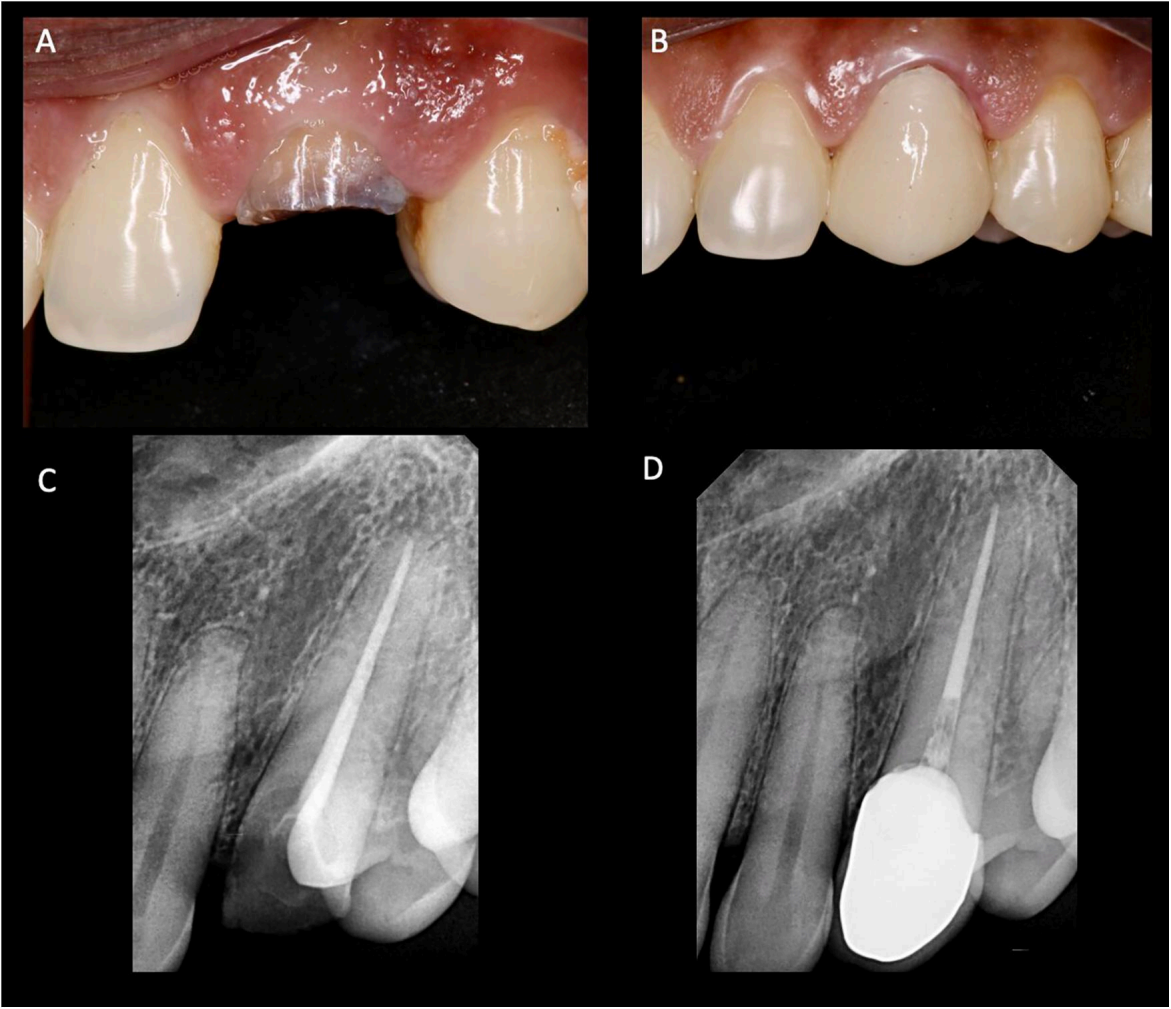
Comparative preoperative and postoperative clinical and radiographic outcomes. **(A)** Preoperative clinical view. **(B)** Postoperative clinical view after definitive rehabilitation. **(C)** Preoperative intraoral periapical radiograph. **(D)** Postoperative intraoral periapical radiograph showing satisfactory post placement and definitive restoration.

Comparative preoperative and postoperative clinical and radiographic evaluation demonstrated successful rehabilitation of the structurally compromised tooth with satisfactory functional and esthetic outcomes (Figures 8A–D).

### Patient perspective

The patient expressed satisfaction with the esthetic outcome and functional rehabilitation achieved following treatment.

### Informed consent

Written informed consent was obtained from the patient for treatment procedures and publication of clinical photographs and radiographic records for academic purposes.

## Discussion

Restoration of structurally compromised endodontically treated teeth (ETT) remains a major restorative challenge because of the significant loss of tooth structure, compromised ferrule effect, and altered biomechanical behavior following endodontic therapy (2, 3). The prognosis of ETT is largely influenced by the amount of residual tooth structure and the ability of the definitive restoration to reinforce the weakened tooth while preserving the remaining dentin (4). In the present case, extensive coronal destruction with subgingival extension of the palatal and proximal margins further complicated restorative management and isolation procedures. In the present case, the compromised ferrule and subgingival extension of margins necessitated a conservative adhesive reinforcement strategy aimed at preserving the remaining tooth structure while improving biomechanical stability.

Traditionally, surgical crown lengthening was advocated for management of deep subgingival margins to obtain restorative access and ferrule (10). However, surgical approaches may compromise periodontal support, crown-root ratio, and esthetics, especially in the anterior region (25). Contemporary adhesive restorative concepts therefore favor minimally invasive procedures such as deep margin elevation (DME), which relocates the cervical margin coronally and facilitates adhesive restorative procedures while preserving periodontal architecture (11, 13, 14). In the present case, laser gingivectomy followed by DME allowed adequate exposure of the subgingival margins and improved restorative accessibility without extensive sacrifice of supporting tissues.

The doughnut technique was incorporated during pre-endodontic buildup to improve structural integrity and isolation while preserving canal access (15, 17). This technique has been reported to enhance rubber dam isolation and facilitate adhesive rehabilitation in severely compromised teeth (16). In the present case, the doughnut technique also aided in achieving a stable coronal foundation before post-space preparation.

Immediate dentin sealing (IDS) was performed after complete caries excavation as part of the adhesive rehabilitation protocol. IDS has been shown to improve bond strength, reduce bacterial leakage, and preserve dentin substrate integrity by sealing freshly cut dentin before restorative procedures (19). The use of IDS in conjunction with adhesive restorative protocols may contribute to improved long-term durability of bonded indirect restorations (19, 26).

Selection of an appropriate post system plays a critical role in the rehabilitation of structurally compromised teeth. Conventional metallic and rigid prefabricated posts exhibit a higher elastic modulus than dentin, resulting in unfavorable stress concentration within the radicular dentin and increasing the risk of catastrophic root fracture (2, 4, 27). In contrast, polyethylene fiber–reinforced post systems possess biomechanical properties closer to that of natural dentin and demonstrate improved stress distribution under functional loading (6, 7, 28).

Polyethylene fibers possess a leno-woven multidirectional architecture that facilitates stress dissipation and crack arrest within the restored tooth complex (7, 8). Recent systematic reviews have reported favorable fracture resistance and predominantly repairable fracture patterns compared with conventional post systems (1, 6, 29).

In addition, their adaptable ribbon design allows close adaptation to canal morphology with minimal post-space enlargement, thereby preserving radicular dentin while providing effective reinforcement of structurally compromised endodontically treated teeth (7, 8).

Adhesive cementation protocols also significantly influence the success of fiber-reinforced restorations. Proper etching, bonding, and resin cementation improve adaptation between dentin, fiber reinforcement, and composite core material, thereby contributing to the formation of a biomechanically integrated restorative complex or “monoblock” effect (20, 21). The use of dual-cure resin cement in the present case ensured adequate polymerization within the post space and enhanced retention of the polyethylene fiber post system.

A full-coverage monolithic zirconia restoration fabricated from Cercon XT zirconia (Dentsply Sirona, Bensheim, Germany) was selected as the definitive prosthetic rehabilitation owing to its favorable esthetic properties, fracture resistance, and long-term clinical performance (22, 24). Contemporary zirconia bonding protocols such as the APC concept, involving air-particle abrasion followed by application of zirconia primer prior to adhesive cementation, have been shown to improve bond durability and retention (23). In the present case, adherence to the APC protocol contributed to satisfactory marginal adaptation and postoperative periodontal response observed during follow-up evaluation.

The successful outcome observed in this case highlights the importance of combining conservative adhesive strategies, biomimetic reinforcement concepts, and contemporary restorative protocols in the rehabilitation of structurally compromised endodontically treated teeth (29). The use of deep margin elevation, immediate dentin sealing, doughnut technique, and polyethylene fiber–reinforced post and core allowed preservation of the remaining tooth structure while achieving satisfactory esthetic and functional rehabilitation.

## Conclusion

Rehabilitation of structurally compromised endodontically treated teeth requires a multidisciplinary and biomimetic restorative approach aimed at preserving the remaining tooth structure while ensuring long-term functional and esthetic success. The present case demonstrated that the combined use of laser-assisted margin exposure, deep margin elevation, immediate dentin sealing, doughnut technique, and polyethylene fiber–reinforced post and core can effectively reinforce weakened tooth structure while minimizing additional radicular dentin removal.

Polyethylene fiber reinforcement provided a conservative adhesive alternative to conventional rigid post systems by promoting favorable stress distribution and biomimetic reinforcement of the restored tooth complex. Furthermore, adherence to contemporary adhesive protocols and zirconia surface conditioning procedures contributed to satisfactory clinical adaptation and periodontal healing.

Within the limitations of a single case report, this technique appears to be a promising minimally invasive treatment strategy for the rehabilitation of structurally compromised endodontically treated teeth with subgingival defects. Long-term clinical studies are required to further evaluate the durability and clinical performance of polyethylene fiber–reinforced restorations in such challenging cases.

## Patient consent statement

Written informed consent was obtained from the patient for all clinical procedures and for publication of clinical photographs and radiographic data for academic and scientific purposes. Patient anonymity has been adequately preserved.

## Data Availability

The raw data supporting the conclusions of this article will be made available by the authors, without undue reservation.
